# Genome-wide identification and characterization of circular RNAs for exogenous trehalose-mediated heat stress responses in tea plants (*Camellia sinensis*)

**DOI:** 10.3389/fpls.2024.1481169

**Published:** 2024-12-05

**Authors:** Shizhong Zheng, Chufei Liu, Ziwei Zhou, Liyi Xu, Biyuan Ruan, Xiaohui Chen

**Affiliations:** ^1^ College of Biological Science and Engineering, Ningde Normal University, Ningde, China; ^2^ Institute of Horticultural Biotechnology, Fujian Agriculture and Forestry University, Fuzhou, China; ^3^ Agricultural Products Quality Safety Inspection and Testing Center, Ningde Agricultural and Rural Bureau, Ningde, China

**Keywords:** circular RNAs, heat stress, trehalose, DNA double strand breaks, tea (*Camellia sinensis*)

## Abstract

**Background:**

Heat stress is one of the main environmental factors limiting the growth, yield and quality of tea plants (*Camellia sinensis*). Trehalose involved in plant responses to multiple adverse environmental stresses, including heat stress. However, the roles of circular RNAs (circRNAs) and their involvement in the trehalose response to heat stress remain unknown.

**Methods:**

In this study, circRNA-sequencing was performed to analyze the characteristics of circRNAs in trehalose-induced responses to heat stress in tea plants. Kyoto Encyclopedia of Genes and Genomes enrichment analysis was used to determine the potential function of circRNAs, and the expression of differentially expressed circRNAs (DECs) and their host genes related to Non-homologous end-joining (NHEJ) and Homologous recombination (HR) were analyzed. To further explore the effect of trehalose on DNA double strand breaks (DSBs), the reactive oxygen species (ROS) contents, specially hydrogen peroxide (H_2_O_2_) and superoxide anion (O2^−^), in heat-stressed tea plants were investigated.

**Results:**

A total of 11402 circRNAs were detected from CK, T (heat stress) and TT (heat stress + trehalose) samples. Among these circRNAs, 573, 620 and 550 circRNAs were identified as differentially expressed in the T vs. CK, TT vs. CK and TT vs. T comparison groups, respectively. The host genes of DECs were enriched in NHEJ and HR pathways, implying a critical role of circRNAs in DSBs repair. The expression level of circKu70-1 and circKu70-3 showed positive correlations with their host gene, *ATP-dependent DNA helicase II 70 kDa subunit (CsKu70)*, while circKu70-2 exhibited an opposite expression trend. Similarly, circRad50 displayed a negative correlation with its host gene, *DNA repair protein RAD50 (CsRad50)*. Notably, the expression of *CsKu70* and *CsRad50*, which are crucial for initiating DSB repair, was decreased in the trehalose-treated (TT) samples. This finding suggests that trehalose may play a role in modulating the expression of circRNAs and their host genes involved in NHEJ and HR pathways, ultimately contributing to reduced DSB damage during heat stress. Moreover, exogenous trehalose significantly reduced H_2_O_2_ and O2^−^ contents in tea plants under heat stress, suggesting that trehalose could mitigate heat-induced damage resulting from ROS overproduction.

**Conclusion:**

Our results indicated that circRNAs play a crucial role in maintaining genome integrity. Specifically, they may function as molecular hubs that respond to changes of the levels of H_2_O_2_ and O2^−^ induced by trehalose, and subsequently regulate the DSBs mediated by their host genes. This, in turn, further impacts genome stability, ultimately enhancing heat tolerance in tea plants. Our findings provided new insight into the potential applications of trehalose as an agrochemical in tea plants and revealed the potential role of circRNAs in tea plants heat tolerance.

## Introduction

Tea plant (*Camellia sinensis*) is an important leaf-based economic crop and is widely cultivated in the tropical and sub-tropical regions of the world. Tea is healthy non-alcoholic beverage, which is rich in taste, aroma, flavor, and multiple polyphenolic compounds. However, global warming triggered by the greenhouse effect has posed challenges for tea quality and production. Therefore, it is imperative to develop approaches to enhance heat tolerance of tea plants. One of the strategies is seeking an eco-friendly protective agent to achieve sustainable yield and quality improvement of tea plants.

Trehalose (α-D-glucopyranosyl-1,1-α-D-glucopyranoside) is a nonreducing disaccharide composed of two molecules of glucose. Trehalose is found in diverse organisms and has various effects, including as osmolyte, storage reserve, transport sugar, and stress protectant (Figueroa and Lunn, 2016). In plants, trehalose has been reported to play an important role in plant biological processes, such as embryo development (Eastmond et al., 2002; Gómez et al., 2006), photosynthesis leaf development (Pellny et al., 2004), regulation of stomatal conductance (Van Houtte et al., 2013). Besides, it has been widely documented that trehalose can regulate plant responses to various biotic and abiotic stresses, including rhizobial and mycorrhizal symbioses (Suárez et al., 2008), insect and pathogen defense (Fernandez et al., 2010), copper (Mostofa et al., 2015), salt (Henry et al., 2015), and drought tolerance (Kondrák et al., 2012; Lee et al., 2003). In recent studies, trehalose has been used to protect plants from high-temperature-induced damage. Exogenous application of trehalose confers high-temperature stress tolerance to herbaceous peony (*Paeonia lactiflora* Pall.) by enhancing antioxidant systems, activating photosynthesis, and protecting cell structure (Zhao et al., 2019). Moreover, exogenously supplied trehalose protects thylakoid membranes and the photosynthetic capacity of winter wheat (*Triticum aestivum*) from heat-induced damage, and decreases electrolyte leakage, malondialdehyde (MDA) content, superoxide anion (O_2_^−^) content, hydrogen peroxide (H_2_O_2_) content, and lipoxygenase activity (Luo et al., 2010). Furthermore, it is also reported that the tolerance to heat stress in tea plants induced by trehalose is attributed to the enhanced activity of antioxidant enzymes, such as superoxide dismutase and peroxidase (Zheng et al., 2024).

Circular RNAs (circRNAs) are single-stranded RNAs processed from their cognate linear RNAs where a downstream 5’ splicing donor site is joined to an upstream 3’ splicing acceptor site (Ashwal-Fluss et al., 2014). Increasing evidence shows that circRNAs play important roles in various developmental processes and environmental stress responses. It has been reported that circRNAs have essential functions in photosynthetic machinery and metabolite biosynthesis during tea leaf development (Tong et al., 2018). In addition, it is reported that circRNAs are involved in regulating cold tolerance in tea plants (Huang et al., 2023). Moreover, circRNAs serve as effective indicators of drought responses in Arabidopsis and maize (*Zea mays*) (Zhang et al., 2019). Genome-wide identification of cucumber (*Cucumis sativus*) circRNAs revealed that large number of circRNAs play essential roles in response to salt stress (Zhu et al., 2019). Furthermore, heat stress has been found to enhance the accumulation and alter the genome-wide profiles of circRNAs in Arabidopsis (Pan et al., 2018). However, the role of circRNAs in regulating tea plants under heat stress remains unclear. Additionally, the mechanisms of circRNAs in tea plants’ enhanced tolerance to heat stress induced by trehalose treatment are still elusive.

In this study, the tea plants (*C.sinensis* cv. Tieguanyin) were subjected to heat stress and recorded after 48 h heat treatment. Then, the second and third leaves from the top of tea plants were collected at 0 h (CK), after 24 h of heat stress with water (T), and 24 h of heat stress with 5 mM trehalose (TT) treatment, respectively. A genome-wide identification of circRNAs were performed in CK, T and TT samples in order to fully understand the potential of function of circRNAs in tea plants under trehalose-induced enhanced tolerance to heat stress. In addition, to explore the effect of trehalose on reactive oxygen species (ROS) metabolism, we analyzed the H_2_O_2_ and O_2_^−^ changes among CK, T and TT samples. Our study provides new insights into the role of trehalose in tea plants’ heat tolerance and lays a foundation for further functional studies of tea circRNAs’ response to heat stress.

## Materials and methods

### Plant materials and treatments

Two-year-old tea plants (*C.sinensis* cv. Tieguanyin) were placed in an artificial climate incubator with 12 h light (25 °C)/12 h dark (19°C) and 75% relative humidity. After 7 d of adaptive growth, the tea plants were randomly divided into three groups and incubated at 12 h light (38°C)/12 h dark (29°C), and 75% relative humidity for heat stress treatment. It is worth mentioning that heat stress was recorded after 48 h of heat pretreatment. Then, tea plants in different groups were treated as follows: (1) CK (Negative Control): tea leaves were sprayed with distilled water (ddH_2_O) and the leaves were collected immediately (at 0 h); (2) T (Positive Control): tea leaves were sprayed with ddH_2_O and the leaves were collected after 24 hours (at 24 h); (3) TT (Trehalose-Treated Group): tea leaves were sprayed with 5 mM trehalose and the leaves were collected after 24 hours (at 24 h). In our previous study, we investigated the protective effect of trehalose on tea plants subjected to heat stress. By comparing different concentrations of trehalose (2.5, 5.0, and 10.0 mM), we found that 5 mM trehalose was particularly effective in alleviating the damage caused by 24 h heat stress in tea plants. In terms of phenotypic effects, the control group of tea plants exposed to heat stress exhibited notable defoliation and dead tissues. In contrast, the tea plants treated with 5.0 mM trehalose displayed a healthier overall appearance, with less defoliation and fewer dead tissues compared to the control group (Zheng et al., 2024). The second and third leaves from the top of the tea plant were used in this study. Three biological replicates were collected for each sample. All the leaf samples were frozen in liquid nitrogen and then stored at -80°C for subsequent analysis.

### Determination of hydrogen peroxide and superoxide anion contents

The hydrogen peroxide (H_2_O_2_) content was determined using an H_2_O_2_ kit from Grace Biotechnology (Suzhou, China). In brief, 0.1 g of each tea leaf sample was ground in an ice bath with 1 mL of acetone. After centrifugation at 12000 rpm for 10 min at 4°C, the absorbance of the supernatant was quantified at 415 nm using a SpectraMax 190 spectrophotometer (Molecular Devices, CA, USA). Finally, the H_2_O_2_ concentrations of tested samples were determined based on a H_2_O_2_ standard curve.

The superoxide anion (O_2_^−^) content was determined using an oxygen free radical kit from Grace Biotechnology (Suzhou, China). Briefly, 0.1 g of tea leaves of CK, T and TT samples was homogenized in 1 mL of extraction solution in an ice bath. After a centrifugation at 12000 rpm for 10 min at 4°C, the absorbance of the supernatant was measured at 540 nm following the manufacturer’s protocol. The O_2_^−^ content was calculated based on an O_2_^−^ standard curve.

The contents of H_2_O_2_ and O_2_^−^ were measured in triplicate.

### circRNA library construction and sequencing

Total RNA was isolated from CK, T and TT samples using the mirVana miRNA Isolation Kit (ThermoFisher Scientific, MA, USA). RNA integrity was evaluated using the Agilent 2100 bioanalyzer (Agilent technologies, Santa Clara, CA, USA). The samples with an RNA Integrity Number (RIN) ≥ 7 were selected for subsequent analysis. Total RNA (1 μg) was depleted of ribosomal RNAs (rRNA) using the TruSeq Stranded Total RNA LT with Ribo-Zero Plant (Illumina, San Diego, CA, USA). The rRNA-depleted RNAs were then treated with 3 units/μg of RNase R (Epicentre, Shanghai, China) at 37°C for 15 min. First-strand complementary DNA (cDNA) was synthesized using SuperScript II Reverse Transcriptase (Thermo) with randomized hexamer. The purified cDNA strand undergoes end repair and 5’ adaptor ligation, followed by fragment size selection and PCR amplification. Finally, the qualified libraries were sequenced on the Illumina sequencing platform (HiSeq 2500) and paired-end reads were generated by Shanghai OE Biotech.

### Identification and quantification of circRNAs

For genome-wide identification of circRNAs, low-quality reads were removed, including reads with adaptor sequences, reads with > 5% N, or > 20% bases with quality < Q20 (percentage of sequences with sequencing error rates < 1%). The resulting clean reads were then mapped to the Tieguanyin genome (Zhang et al., 2021). In this study, find_circ was used to identify potential circRNAs. Find_circ extracted circRNAs by recognizing back-splice sites. Reads that aligned continuously to the genome were discarded. Next, the terminal parts of the unmapped reads were extracted and mapped to the genome. Anchor positions arranged in the opposite direction of the reference sequence were selected. Finally, the sequences between the alignments were extended and circRNAs were identified. BEDTools was used to identify the positional relationship between circRNAs and adjacent coding RNAs (Quinlan, 2014). Based on their genomic origins, circRNAs can be classified as exonic (originating from exons), intronic (originating from introns), intergenic (originating from intergenic regions), antisense (transcribed from the opposite strand of their gene locus and overlapping with linear RNA), and sense-overlapping (originating from the same gene locus but not belonging to the “exonic” or “intronic” categories) (Liu et al., 2017).

For the quantification of circRNAs, the relative expression levels of the circRNAs were normalized using junction reads per billion clean reads (RPB) algorithm based on the number of junction reads spanning transcripts per billion clean reads. Differentially expressed circRNAs (DECs) between sample groups was determined by DEGseq (Anders and Huber, 2012). The circRNAs with a fold-change ≥ 2.00 and a Q-value ≤ 0.05 were identified as significantly DECs.

### Validation and quantitative real-time PCR of circRNAs

To validate circRNAs, genomic DNA or cDNA was used as templates in PCR amplification. Genomic DNA was extracted from tea leaves using the cetyltrimethylammonium bromide method (Li et al., 2013). First-strand cDNA was synthesized with random hexamer primers using a RevertAid First Strand cDNA Synthesis Kit (Thermo). Divergent and convergent primers were designed using Primer 3.0.1 software (https://bioinfo.ut.ee/primer3/) for circRNA validation (**Supplementary Table S1**). Divergent primers were used to amplify candidate circRNAs. For controls, convergent primers were used to detect linear mRNAs. PCR was carried out using a Bio-RadT100 Thermocycler with the following procedure: 95°C for 30 s per cycle; 30 cycles of 95°C for 30 s, 60°C for 20 s and 68°C for 30 s; and then 68°C for 5 min per cycle. The PCR products were separated by agarose gel electrophoresis and purified with a GeneJET Gel Extraction Kit (Thermo). Sanger sequencing was performed to further confirm the junction sites.

For quantitative real-time PCR (qRT-PCR), the relative expression levels of circRNAs and genes were normalized using *glyceraldehyde-3-phosphate dehydrogenase* (*GAPDH*) and analyzed using the 2^-ΔΔCT^ method. All qRT-PCR analyses were repeated three times. The primers used for qRT-PCR are listed in **Supplementary Table S1**.

### Statistical analysis

Physiological data and qRT-PCR results were analyzed using ANOVA, and differences among groups were further examined using Duncan’s *post hoc* test (at a significance level of P < 0.05) with SPSS 25 software.

## Results

### Genome-wide identification and characteristic analysis of circRNAs in tea plants

**Figure 1:**
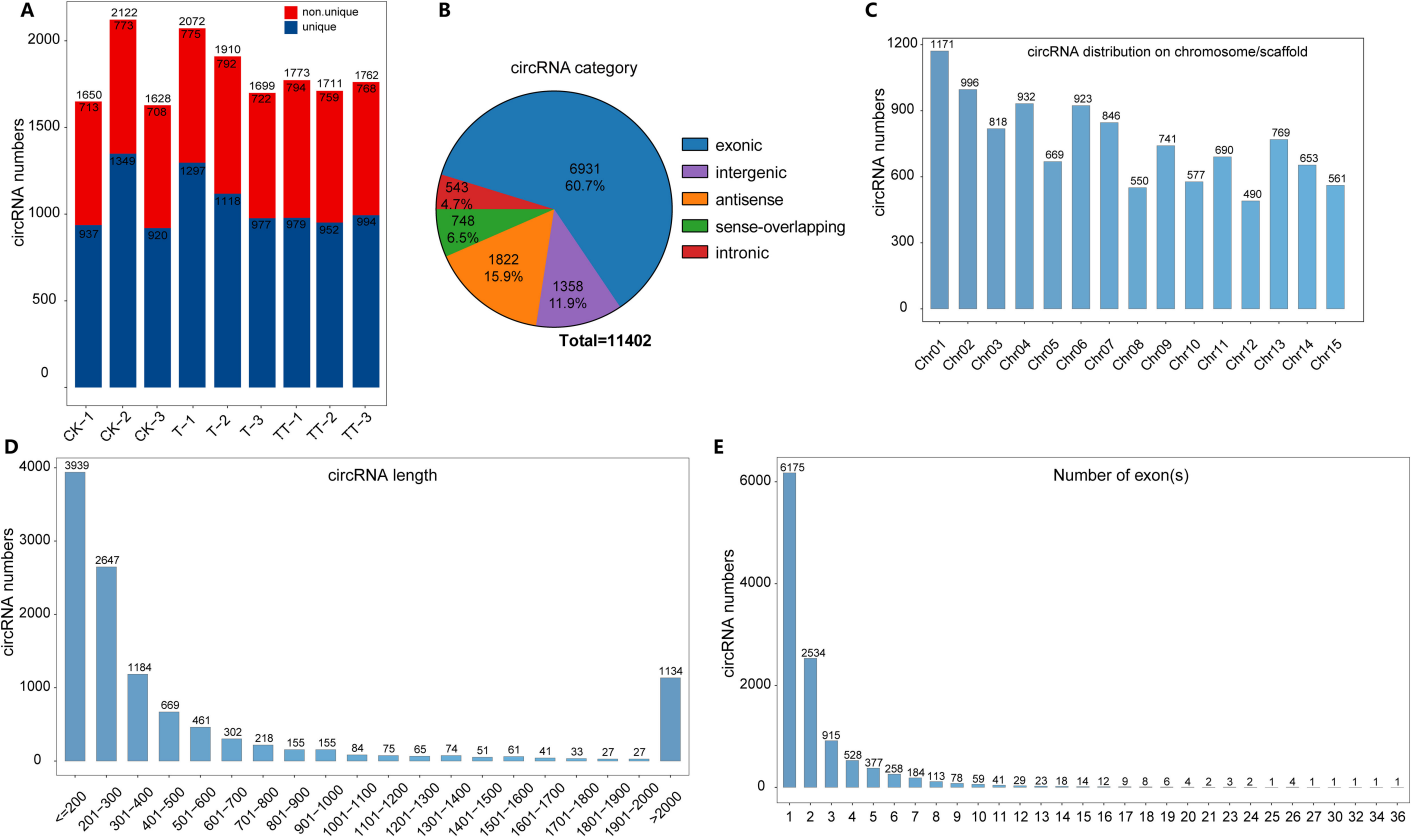
Identification and characteristics of circRNAs in tea leaves. **(A)** Number of circRNAs identified in CK, T and TT from three independent replicates. The red and blue colors represent number of shared and unique circRNAs in different samples, respectively. **(B)** Classification of circRNA according to their position with adjacent coding RNA. **(C)** Distribution of circRNAs in 15 tea chromosomes. **(D)** Length distribution of circRNAs in tea plants. **(E)** Exon number of circRNAs in tea plants.

To investigate the characteristics of circRNAs in tea plants under heat stress regulated by exogenous trehalose treatment, the circRNAs libraries for tea leaves under CK, T and TT different treatments were constructed. After filtering the low-quality reads and adapters sequences, an average of 114.54 M clean reads were generated from nine samples (CK-1, CK-2, CK-3, T-1, T-2, T-3, TT-1, TT-2 and TT-3)(**Supplementary Table S2**). Then, the clean reads were aligned to the Tieguanyin reference genome (Zhang et al., 2021). And find_circ was used to detect circRNAs. Based on the back-splice sites, a total of 1650, 2122, 1628, 2072, 1910, 1699, 1773, 1711 and 1726 circRNAs were predicted in CK-1, CK-2, CK-3, T-1, T-2, T-3, TT-1, TT-2 and TT-3, respectively (**Figure 1A**). A total of 11402 unique circRNAs were detected in tea leaves from these nine samples (**Supplementary Table S3**). According to the position relationship between circRNAs and adjacent coding RNA, circRNAs were classified into five categories: exonic, intronic, intergenic, antisense and sense-overlapping. Among these 11402 circRNAs, exonic (60.7%) was the predominant circRNAs type, followed by antisense circRNAs (15.9%) and intergenic circRNAs (11.9%), while intronic circRNAs (4.7%) had the lowest proportion in tea leaves (**Figure 1B**). The distribution of circRNAs in each chromosome showed that the majority of circRNAs were enriched in chromosome 1, followed by chromosomes 2, 4 and 6 (**Figure 1C**). Additionally, analyses of the circRNA length and exon number distributions showed that most circRNAs were shorter than 300 bp (**Figure 1D**) and most circRNAs contained only one or two exons (**Figure 1E**).

### Validation of circRNAs in tea plants

**Figure 2:**
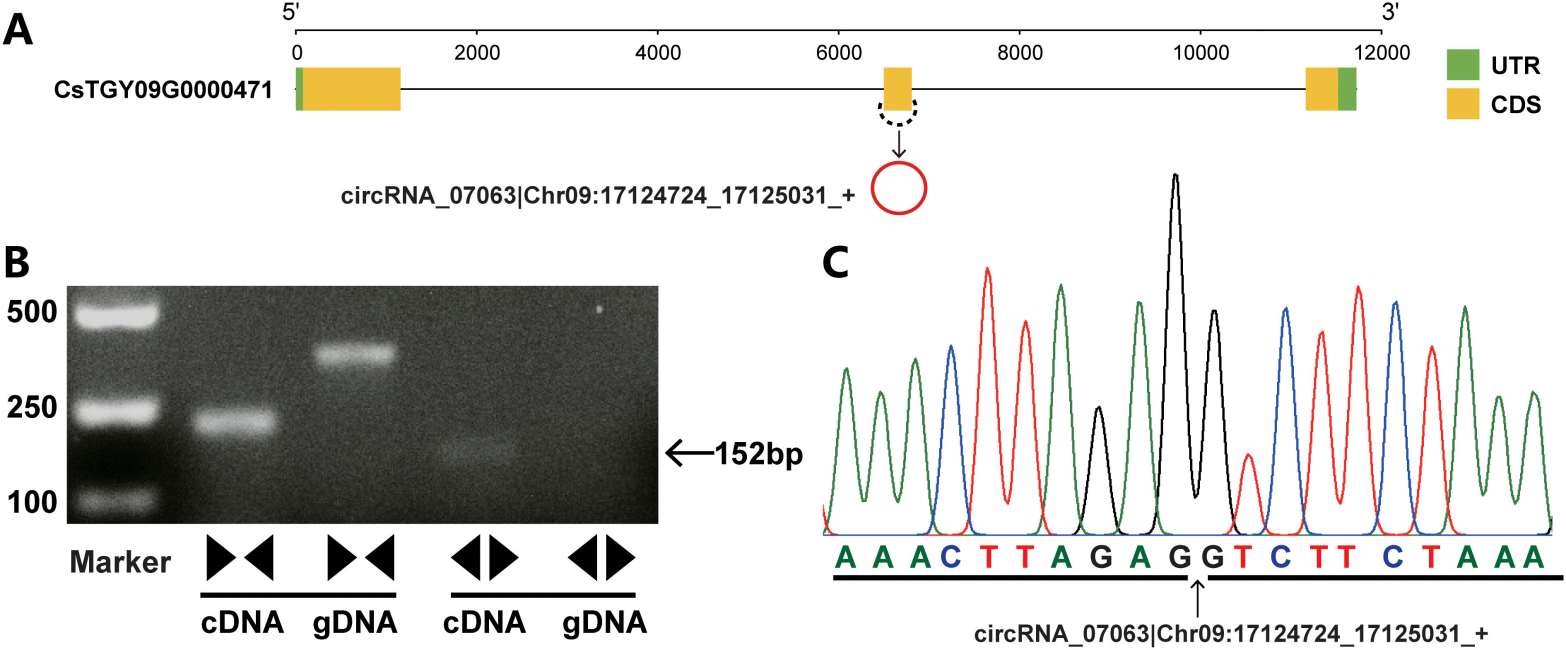
An example of circRNA validation. **(A)** The schematic shows the gene structure of circRNA host gene and circRNA production. The green and yellow boxes indicate the untranslated region (UTR) and coding sequence (CDS), respectively. The black line indicates the introns. circRNAs are shown as red circles. **(B)** Validation of circRNA by PCR amplification with divergent and convergent primers. A pair of convergent primers was used to amplify linear DNA fragments within the cDNA and genomic DNA. By contrast, a pair of divergent primers amplified the fragments from circRNA-derived cDNAs but not genomic DNA. **(C)** The circRNA was confirmed by Sanger sequencing. The arrow indicates the junction sites.

To validate the circRNAs identified by circRNA-seq, 15 exonic circRNAs from the top highly expressed were randomly selected for PCR amplification. A series of convergent and divergent primers were used to amplify the linear RNAs and circRNAs, respectively. A pair of convergent primers could amplify PCR products within the cDNA and genomic DNA. By contrast, a pair of divergent primers only amplified fragments in cDNA but not within the genomic DNA. Sanger sequencing was performed on the fragments generated by the divergent primers to further verify the back-spliced junctions of the circRNAs. The results showed that nine of the 15 exonic circRNAs (60%) validated the presence of back-spliced junctions (**Figure 2**; **Supplementary Figure S1**). In addition, the expression levels of circRNAs were determined by qRT-PCR. The results showed that expression trends of these circRNAs were similar with the circRNA-seq data. Moreover, a melting curve was determined for the circRNA products, which showed that only a single target was amplified during the reaction suggesting the accuracy of circRNAs identified in this study (**Supplementary Figure S2**).

### Identification of differentially expressed circRNAs in tea plants

**Figure 3:**
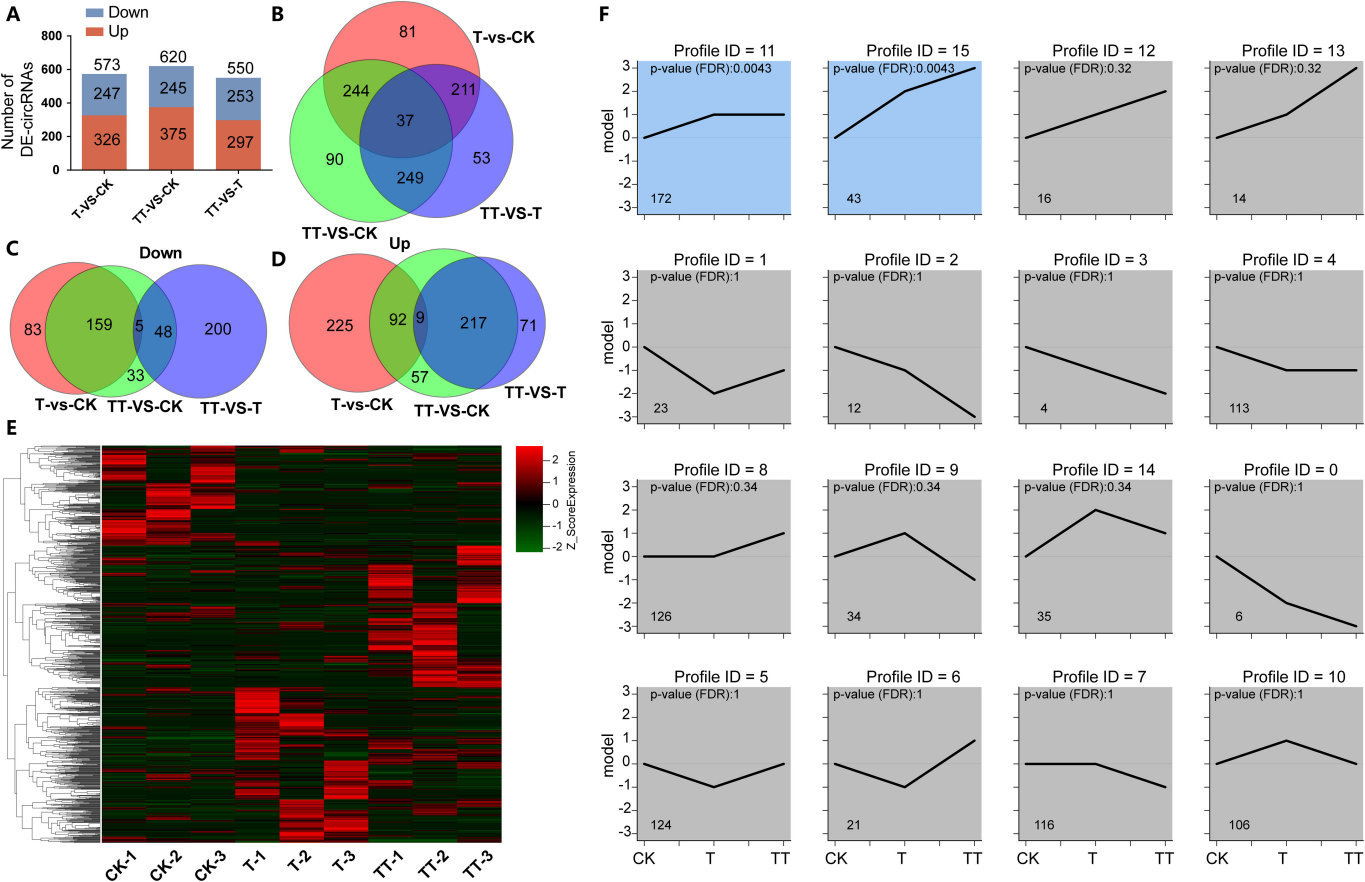
Characteristic of DECs in tea leaves subjected to heat stress as influenced by exogenous trehalose. **(A)** Numbers of DECs among comparison groups of T vs. CK, TT vs. CK and TT vs. T. **(B)** Venn diagram showing the overlap of DECs among T vs. CK, TT vs. CK and TT vs. T. **(C)** Venn diagram showing the overlap of down-regulated DECs among T vs. CK, TT vs. CK and TT vs. T. **(D)** Venn diagram showing the overlap of up-regulated DECs among T vs. CK, TT vs. CK and TT vs. T. **(E)** The Clustered heatmap showing DECs expression pattern in CK, T and TT. **(F)** Expression trend lines of DECs in CK, T and TT samples.

To explore the effect of exogenous trehalose on changes in circRNAs expression in tea plants under heat stress, differentially expressed circRNAs (DECs) among T vs. CK, TT vs. CK and TT vs. T comparison groups were analyzed. The results showed that 573, 620 and 550 DECs were identified in the T vs. CK, TT vs. CK and TT vs. T comparison groups, respectively (**Figure 3A**; **Supplementary Table S4**); among which 37 DECs overlapped (**Figure 3B**). Venn diagrams analysis of down-regulated and up-regulated DECs showed that more down-regulated DECs overlapped between the T vs. CK and TT vs. CK comparison groups (**Figure 3C**), while TT vs. CK and TT vs. T comparison groups had more common up-regulated DECs (**Figure 3D**). The clustered heatmap of DECs expression in CK, T and TT samples suggested that circRNAs in a specific expression pattern responsive to heat stress and exogenous trehalose-induced tolerance to heat stress (**Figure 3E**). Furthermore, the expression trend lines of 965 DECs were analyzed based on their RPB values. The results showed that DECs were divided into 16 groups, and profiles 11 and 15 were significantly (p< 0.01) enriched, suggesting circRNAs were involved in the heat stress response of tea plants under exogenous trehalose treatment (**Figure 3F**).

### Gene ontology and Kyoto encyclopedia of genes and genomes pathway analyses of DEC host genes in tea plants

To investigate the potential role of circRNAs in trehalose-induced tolerance to heat stress, we performed Gene Ontology (GO) and Kyoto Encyclopedia of Genes and Genomes (KEGG) enrichment analyses of DEC host genes. In this study, the clusterProfiler package (version 3.16.0) in R was utilized to conduct both GO and KEGG enrichment analyses, with statistical significance determined at q < 0.05 and p < 0.05 (Yu et al., 2012). There were 865, 887 and 885 DEC host genes in the T vs. CK, TT vs. CK and TT vs. T comparison groups, respectively.

GO enrichment analysis showed that these DEC host genes in the three comparisons groups (T vs. CK, TT vs. CK and TT vs. T) were assigned to the biological process (BP), cellular component (CC), and molecular function (MF) categories. In the T vs. CK comparison, the top three BP GO terms were “starch catabolic process”, “protein maturation” and “DNA recombination”. In CC, DEC host genes involved in “chloroplast starch grain”, “thylakoid lumen”, and “nuclear chromosome, telomeric region” were enriched. In MF, “protein binding involved in protein folding”, “serine-type peptidase activity” and “isoamylase activity” were the three most enriched subgroups. In the TT vs. CK comparison, “megagametogenesis”, “base-excision repair” and “double-strand break repair via non-homologous end joining” were the three most enriched subgroups in BP; In CC, GO terms relative to the “spindle microtubule”, “HAUS complex” and “integral component of endoplasmic reticulum membrane” were enriched; In MF, “protein binding involved in protein folding”, “damaged DNA binding” and “magnesium ion transmembrane transporter activity” were the top three enriched GO terms. In TT vs. T comparison, DEC host genes involved in “magnesium ion transport”, “DNA recombination” and “starch metabolic process” were enriched. In CC, GO terms relative to the “exosome (RNase complex)”, “early endosome” and “cytoplasmic exosome (RNase complex)” were enriched. In MF, the top three terms were “magnesium ion transmembrane transporter activity”, “glycogen (starch) synthase activity” and “starch synthase activity” (**Supplementary Figure S3**).

**Figure 4:**
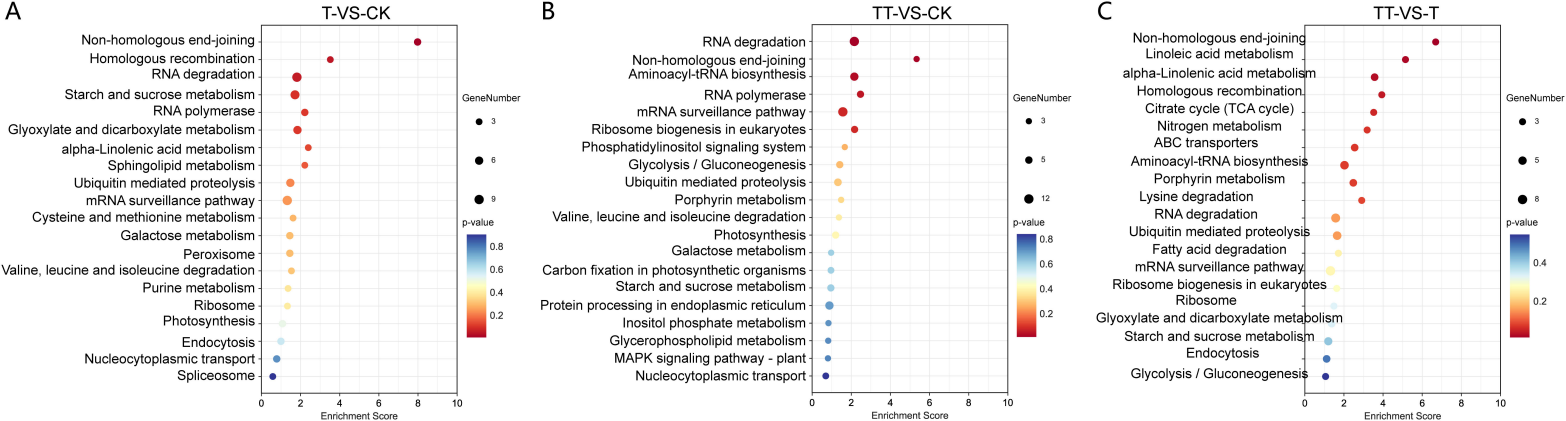
Kyoto Encyclopedia of Genes and Genome enrichment (KEGG) analysis of DEC host genes identified in T vs. CK **(A)**, TT vs. CK **(B)** and TT vs. T **(C)** comparison groups. The higher enrichment score represents more significant enrichment; the lower q-value represents more reliable enrichment. The size of the circle corresponds to gene numbers.

With respect to the KEGG analysis, the results revealed that on the top 20 enriched KEGG pathways, “Non-homologous end-joining (NHEJ)” and “Homologous recombination (HR)” were significant enriched in T vs. CK; “RNA degradation”, “NHEJ”, “Aminoacyl-tRNA biosynthesis” and “RNA polymerase” were significant enriched in TT vs. CK; and “NHEJ”, “Linoleic acid metabolism”, “alpha-Linolenic acid metabolism” and “HR” were significant enriched in TT vs. T (P<0.05) (**Figure 4**).

It is worth noting that “double-strand break repair via non-homologous end joining” was the common GO term enriched in all three comparison groups (T vs. CK, TT vs. CK and TT vs. T). Similarly, “NHEJ” was the shared KEGG pathway enriched in all three comparison groups, suggesting NHEJ plays an important role in tea plants’ response to heat stress.

### Analysis of DECs and their host genes involved in the NHEJ and HR

**Figure 5:**
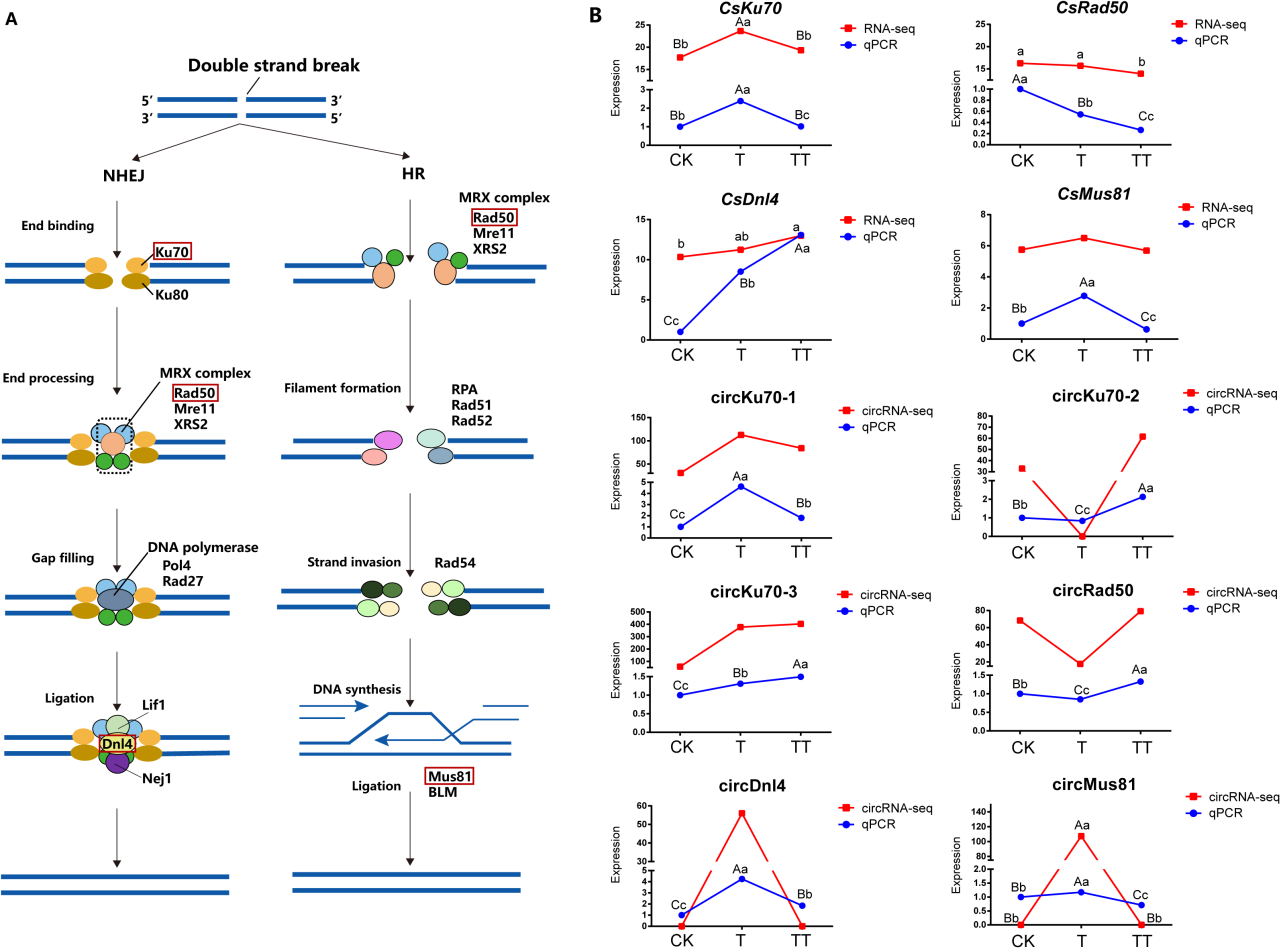
Expression analysis of DECs and their parental genes related to the NHEJ and HR pathways. **(A)** Schematic diagram illustrating the NHEJ and HR pathways. **(B)** The expression patterns of DECs and their parental genes in CK, T and TT samples. The red line graph represents relative expression levels in transcripts, while the blue line graph represents qRCR results. Lowercase letter indicates significant difference (p < 0.05); uppercase letter indicates highly significant difference (p < 0.01).

Based on the KEGG enrichment results, NHEJ was the common enriched pathway in the T vs. CK, TT vs. CK and TT vs. T comparison groups. In addition, HR pathway was found in T vs. CK and TT vs. T comparisons. Both NHEJ and HR pathways are involved in DNA double-strand breaks (DSBs). Therefore, in this study, we focused our attention to the host genes of circRNAs that were involved in NHEJ and HR pathways in this study (**Figure 5A**).

A total of five DECs and three DEC host genes were identified in the NHEJ pathway, while two DECs and two DEC host genes were assigned to HR pathway. Among these DECs and DEC host genes, circKu70-1, circKu70-2 and circKu70-3 corresponded to the gene *CsKu70*; *CsRad50*, *CsDnl4* and *CsMus81* were the host genes of circRad50, circDnl4 and circMus81, respectively. To further investigate the roles of DECs related to the NHEJ and HR pathways in tea plants, the expression levels of these DECs and their host genes were analyzed. The expression levels of these DECs and their host genes were measured by their normalized FPKM and RPB values, respectively. Additionally, the transcript level of DECs and their host genes were confirmed by qRT-PCR (**Figure 5B**; **Supplementary Figure S4**), the results showed that the profiles of these DECs and their parental genes were consistent with the sequencing data.

The results showed that the transcript levels of *CsKu70* and *CsMus81* were higher in the T sample than in the CK and TT; there was a decrease in transcript levels of *CsRad50* in CK, T and TT samples, while *CsDnl4* presented the higher transcript levels in TT than in the other samples. The expression levels of circKu70-1 followed the same trends as its host gene *CsKu70* with higher expression in the T sample compared to the CK and TT samples. In contrast, circKu70-2 showed opposite expression trends compared to *CsKu70*. Moreover, the expression level of circKu70-3 increased in the TT sample compared to the T sample, while *CsKu70* decreased. These results suggested that the circRNAs from the same gene can have different expression trends, and regulatory relationship between circRNA and their parental gene can be positively and negatively correlated (**Supplementary Table S5**). The transcript level of circRad50 was significant lower in the T sample compared to the other samples. The expression level of circDnl4 was significantly increased in the T sample compared to the CK and TT samples, while its parental gene *CsDnl4* presented higher transcript levels in TT than in the other samples. circMus81 and *CsMus81* had similar expression profiles, both being more highly expressed in the T sample compared to the CK and TT (**Figure 5B**).

### Effects of exogenous trehalose on H_2_O_2_ and O_2_^−^contents in tea plants

**Figure 6:**
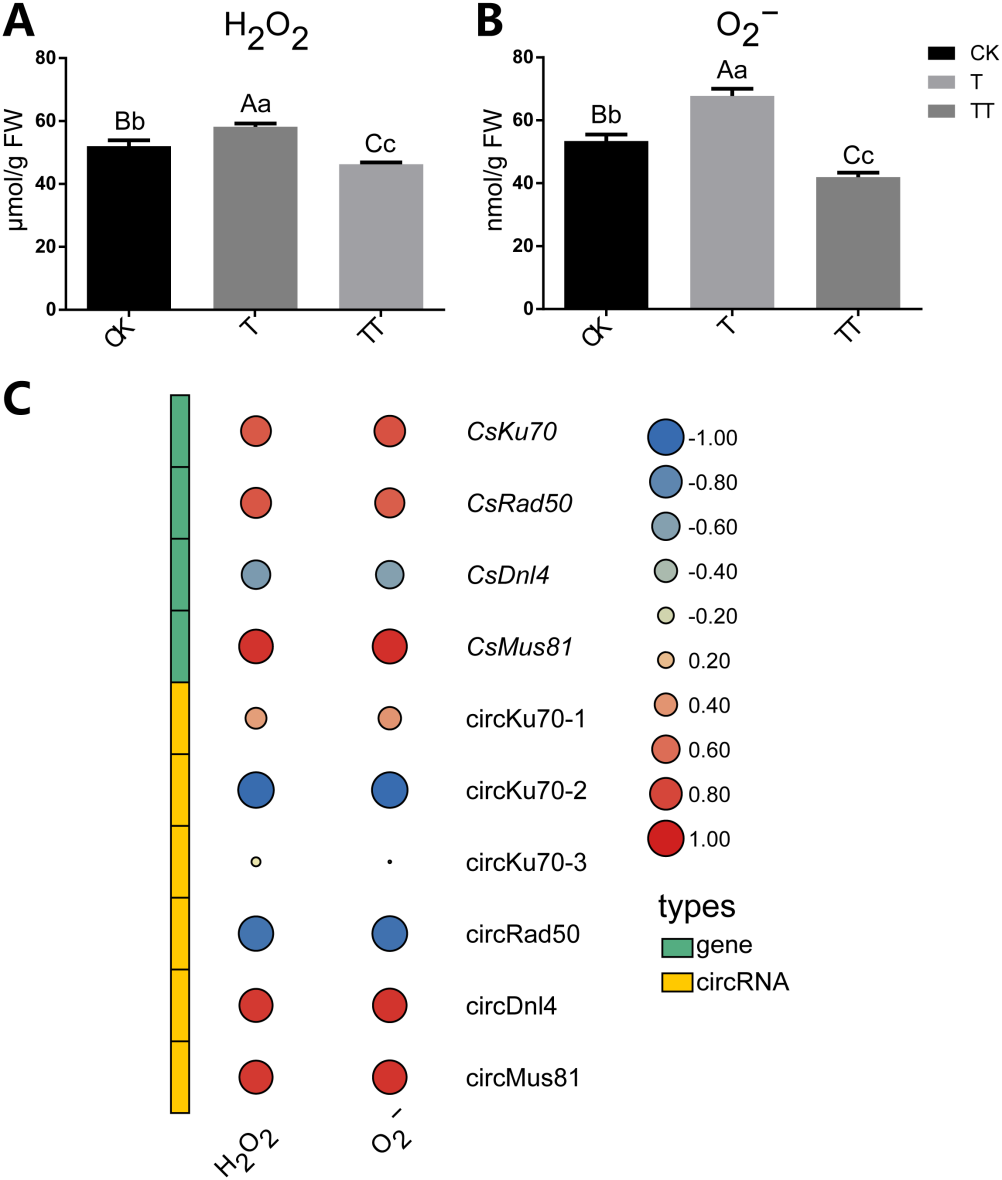
Effects of exogenous trehalose on H_2_O_2_ and O_2_^−^ concentrations under heat stress in tea plants and correlations between the contents of H_2_O_2_ and O_2_^−^ and circRNAs, as well as their host genes that involve in the NHEJ and HR pathways. **(A)** Content of H_2_O_2_. **(B)** Content of O_2_^−^. **(C)** Correlations among circRNAs, circRNA host genes and the contents of H_2_O_2_ and O_2_^−^. Error bars indicate means ± standard deviation (SD). Different normal letters denote significant differences based on Duncan’s *post hoc* test. Lowercase letter indicates significant difference (p < 0.05); uppercase letter indicates highly significant difference (p < 0.01).

To explore the effect of trehalose on oxidative stress in tea plants under heat treatment, we determined the H_2_O_2_ and O_2_^−^ contents, which are considered the primary ROS species. The results showed that heat stress increased H_2_O_2_ and O_2_^−^ accumulation in the tea plants (T sample), while trehalose treatment significantly reduced the H_2_O_2_ and O_2_^−^ contents in heat-treated tea plants (TT sample) (**Figures 6A, B**). These findings suggested that trehalose treatment alleviated heat-induced ROS stress in tea leaves.

Next, we calculated the correlations between the contents of ROS and circRNAs, as well as their host genes that involve in the in the NHEJ and HR pathways (**Figure 6C**). The results showed that circKU70-1 was negatively correlated with H_2_O_2_ and O_2_^−^ contents, while its host gene *CsKu70* was positively correlated with H_2_O_2_ and O_2_^−^ contents. Furthermore, a similar correlation was observed between circRad50 and its host gene *CsRad50* with respect to H_2_O_2_ and O_2_^−^ contents. Additionally, the expression levels of *CsDnl4* was negatively correlated with H_2_O_2_ and O_2_^−^ contents, while circDnl4 expression was positively correlated with H_2_O_2_ and O_2_^−^ contents. Both *CsMus81* and circMus81were positively correlated with H_2_O_2_ and O_2_^−^ contents. Collectively, these results underscore the involvement of circRNAs and their host genes in the NHEJ and HR pathways, suggesting their crucial roles in mediating the cellular response to ROS.

## Discussion

### circRNAs are involved in trehalose-induced tolerance to heat stress by regulating the NHEJ and HR pathways in tea plants

circRNAs are recognized as a new class of non-coding RNA that is widespread in animals and plants and has substantial regulatory functions (Li et al., 2018). In plants, circRNAs are involved in developmental regulation and stress-induced responses (Ye et al., 2015). Here, a total of 11402 unique circRNAs were identified in tea leaves from CK, T and TT samples (**Figure 1A**; **Supplementary Table S3**), and 573, 620 and 550 circRNAs were found to be differentially expressed in the T vs. CK, TT vs. CK and TT vs. T comparison groups, respectively (**Figure 3A**). In addition, the DECs (profiles 11 and 15) whose expression increased in the T and TT samples were significantly (p< 0.01) enriched (**Figure 3F**). These findings indicated that these circRNAs are purposefully generated and have a specific function in heat responses.

To further explore the potential role of circRNAs in response to heat stress in tea plants, we performed KEGG analysis of DEC host genes in the T vs. CK, TT vs. CK and TT vs. T comparison groups (**Figure 4**). The results showed that the most highly enriched KEGG pathways were NHEJ and HR pathway, suggesting circRNAs play critical roles in these two pathways in response to heat stress. NHEJ and HR are DNA repair pathways that involve ligation of the DSB ends. Ku70/80 heterodimer are key players in NHEJ and bind to ends of broken DNA molecule to initiate DNA repair process. In contrast to NHEJ, HR is initiated by the binding of a MRE11-RAD50-XRS2 (MRX) complex to DNA ends (Weterings and Chen, 2008). Therefore, the abundance of Ku70/80 heterodimer and MRX complex could reflect DNA damage to a certain extent. In this study, the expression of *CsKu70* increased under heat stress (T sample) but decreased under trehalose + heat stress (TT sample). Moreover, the transcript levels of *CsRad50* was lowest in trehalose + heat stress (TT sample) (**Figure 5B**). These findings indicated that exogenous trehalose application reduces damaged DNA in tea plants under heat stress. In addition, the concentration of ROS was significantly decreased in tea plants treated with trehalose under heat-stress (TT sample) compared to the tea plants under only heat-stress (T sample) (**Figure 6**). Taken together, our results suggested that trehalose contributes to reducing DSBs by scavenging heat-induced ROS, and circRNAs are involved in NHEJ and HR pathways to maintain genome stability and enhance heat tolerance in tea plants. In support of this, it has been shown that circRNAs are involved in NHEJ and play an important role in DNA repair and cell cycle regulation during longan embryogenesis (Chen et al., 2022). In addition, a recent study showed that circ-MDM2 may be a p53 and cell cycle progression regulator, suggesting that circRNAs are regulators of DNA damage response and repair network components (Chaudhary et al., 2020).

### Trehalose alleviates heat-induced oxidative stress in tea plants

The heat stress influences physiological, biochemical, and molecular changes in plants, including accumulation of ROS, the alteration of plant hormones levels, and the alteration of the transcriptomic and metabolomic profiles (Hasanuzzaman et al., 2013; Wahid et al., 2007). Excessive generation of ROS leads to oxidative stress, which damages stability of macromolecules (DNA, proteins, and membranes), and produces toxicity in cell (Sairam and Tyagi, 2004). The production of ROS in organisms can reflect the damage and resistance status of plants under adverse environmental conditions. In recent studies, exogenous applications of trehalose have been found to be effective in enhancing heat resistance by scavenging ROS. For instance, trehalose has been shown to scavenge ROS in wheat under heat stress (Luo et al., 2008). Moreover, it has been reported that trehalose accumulation enhances the resistance to oxygen radicals in *Saccharomyces cerevisiae* during heat stress (Benaroudj et al., 2001). Similarly, a previous study showed that yeast cells improved tolerance to H_2_O_2_ when treatment with 10% trehalose (da Costa Morato et al., 2008). Consistent with these previous results, in our study, the concentrations of H_2_O_2_ and O_2_^−^ were significantly increased under heat-stress conditions. However, when exogenous trehalose was applied to tea plants under heat-stress, the content of H_2_O_2_ and O_2_^−^ exhibited a significantly decrease (**Figure 6**). This suggests that trehalose alleviates heat-induced oxidative stress in tea plants. Furthermore, the application of exogenous trehalose in tea plants is an effective approach for defense against heat stress.

## Conclusions

In summary, we identified a large number of circRNAs in tea plants, and hundreds of them were found to be differentially expressed in three comparison groups (T vs. CK, TT vs. CK and TT vs. T), which suggested that these circRNAs are effective indicators of tea plants’ heat stress responses. The most highly enriched KEGG pathways of DEC host genes were NHEJ and HR pathways, indicating that circRNAs may play an important role in maintaining genome stability under heat stress in tea plants. In addition, our findings suggested that trehalose enhances resistance to heat stress by scavenging of ROS in tea plants. Overall, our results provide new insights into the potential applications of trehalose in tea plants, and contribute to understanding of circRNAs regulation in heat stress resistance.

## Data Availability

The datasets presented in this study can be found in online repositories. The names of the repository/repositories and accession number(s) can be found below: BioProject, PRJNA1178683.

## Supplementary material

Supplementary Table 1 Summary of primers used in this study.

Supplementary Table 2 Summary of circRNA-seq data after filtering.

Supplementary Table 3 The list of circRNAs identified in this study.

Supplementary Table 4 Differentially expressed circRNAs in the three pairwise comparisons of T *vs.* CK, TT vs. CK and TT vs. T.

Supplementary Table 5 Correlation analysis of circRNAs and their host genes.

Supplementary Figure 1
**(A)** Electrophoretogram showing validation of circRNA by PCR amplification with divergent and convergent primers. **(B)** Validation of circRNAs by Sanger sequencing. The red and green colors indicate the gene structure of circRNA host genes. The bottom panel shows a Sanger sequencing result highlighting the junction site of circRNAs. The scissors symbol indicates the junction sites.

Supplementary Figure 2
**(A)** Expression patterns of circRNAs in RNA-Seq and qPCR for CK, T and TT samples. **(B)** Melting curve analysis of qPCR products to verify amplification of a single target.

Supplementary Figure 3 GO enrichment analysis of DEC host genes identified in T vs. CK **(A)**, TT vs. CK **(B)**, and TT vs. T **(C)**.

Supplementary Figure 4 Melting curve analysis of qPCR products to verify amplification of a single target in NHEJ and HR pathways.
