# Supplementary figures and images for: Genome-wide identification and characterization of circular RNAs for exogenous trehalose-mediated heat stress responses in tea plants (Camellia sinensis)
Source: Front Plant Sci. 2024 Dec 5;15:1481169. doi: 10.3389/fpls.2024.1481169 (PMC11655237; doi:10.3389/fpls.2024.1481169)

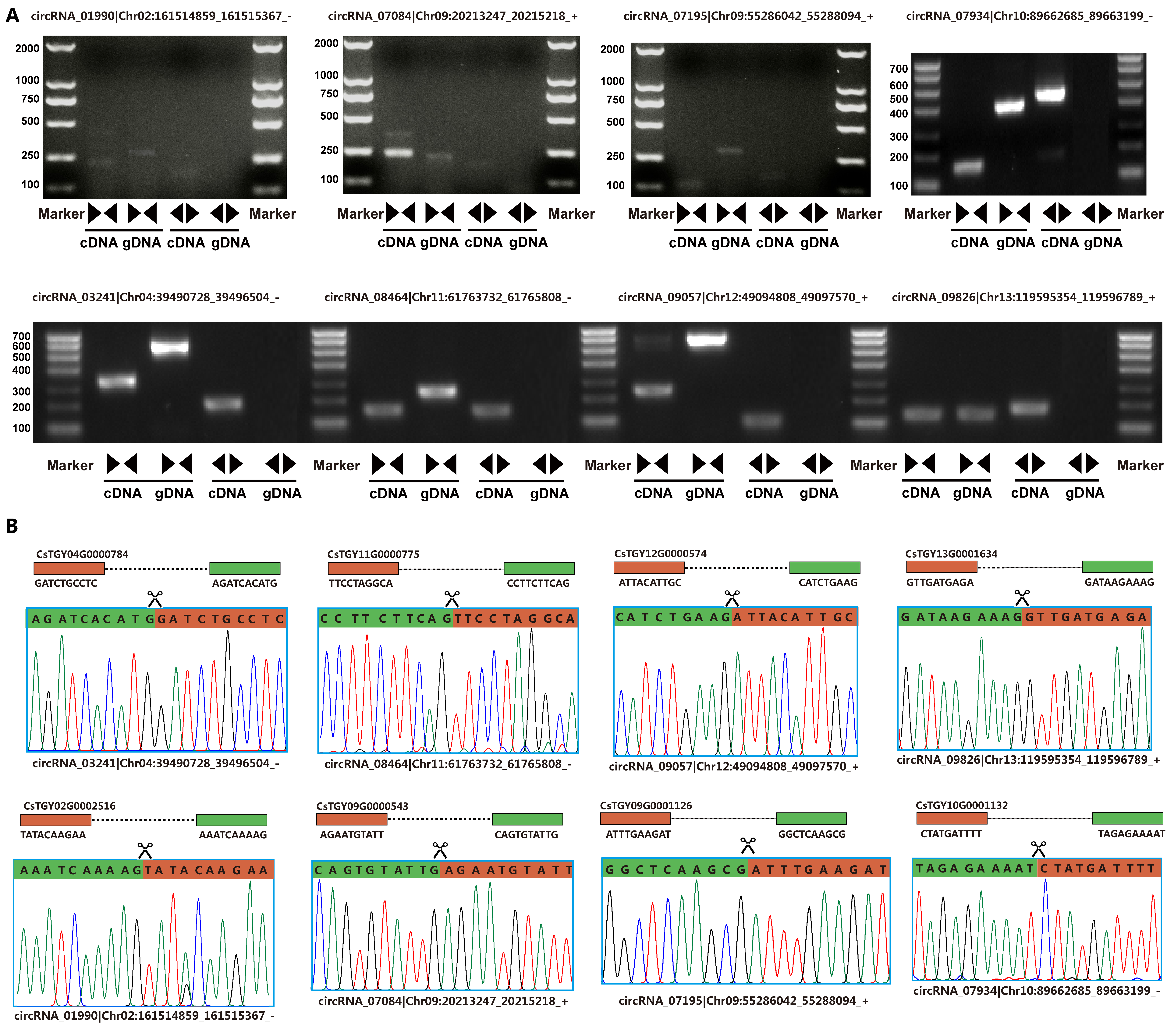

Supplement: Supplementary Figure 1 — (A) Electrophoretogram showing validation of circRNA by PCR amplification with divergent and convergent primers. (B) Validation of circRNAs by Sanger sequencing. The red and green colors indicate the gene structure of circRNA host genes. The bottom panel shows a Sanger sequencing result highlighting the junction site of circRNAs. The scissors symbol indicates the junction sites. [file Image1.tif]

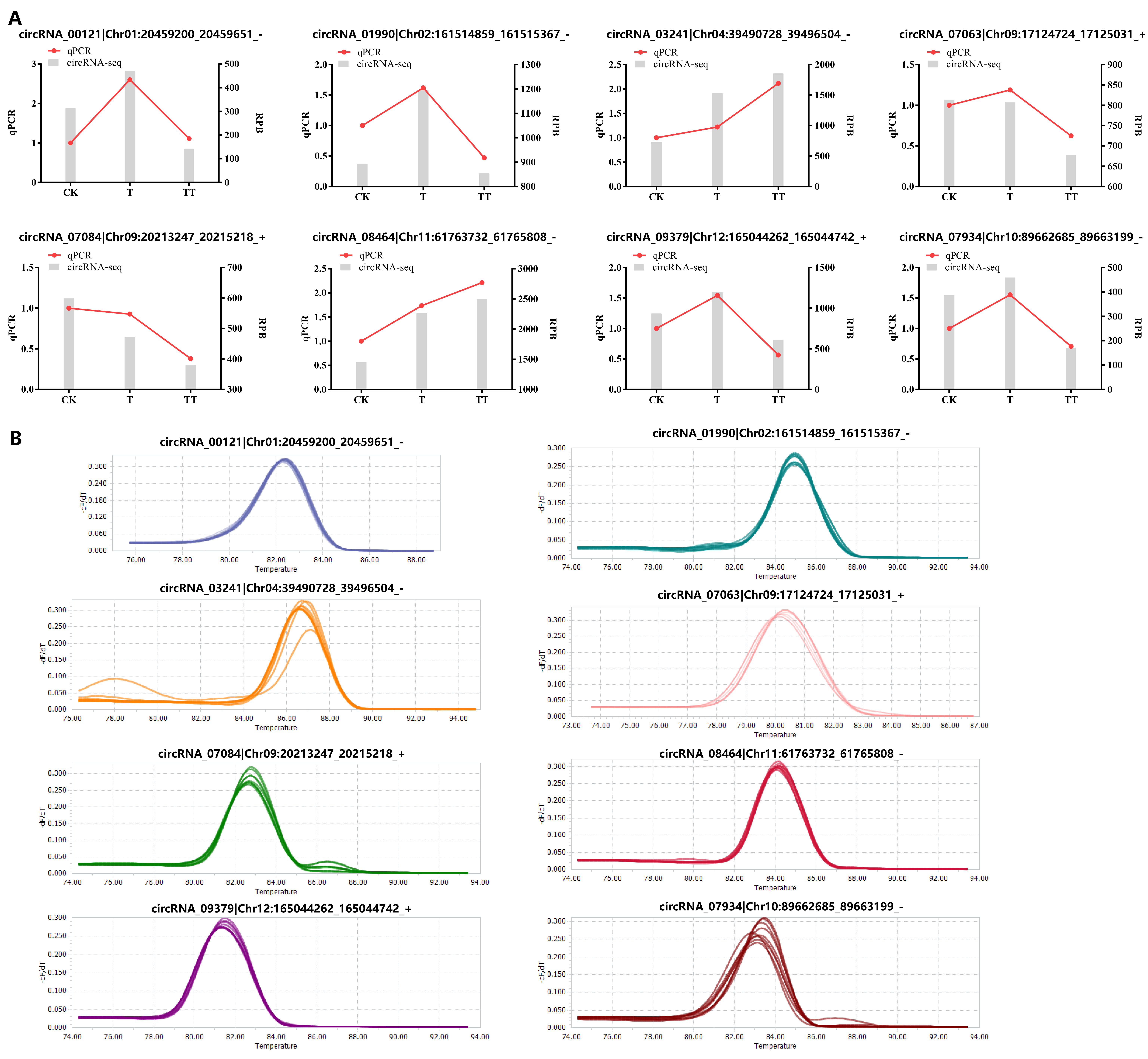

Supplement: Supplementary Figure 2 — (A) Expression patterns of circRNAs in RNA-Seq and qPCR for CK, T and TT samples. (B) Melting curve analysis of qPCR products to verify amplification of a single target. [file Image2.tif]

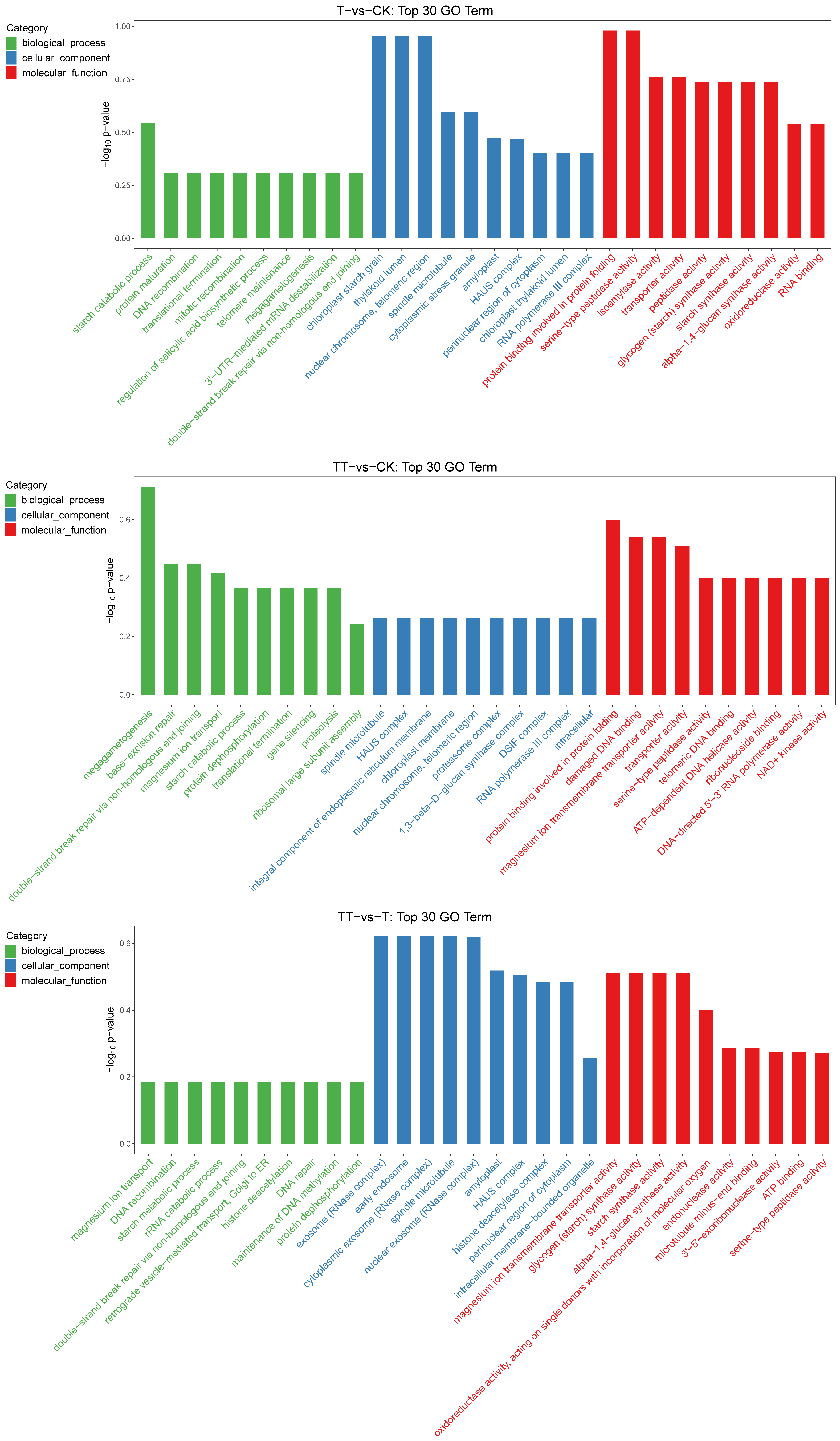

Supplement: Supplementary Figure 3 — GO enrichment analysis of DEC host genes identified in T vs. CK (A), TT vs. CK (B), and TT vs. T (C). [file Image3.tif]

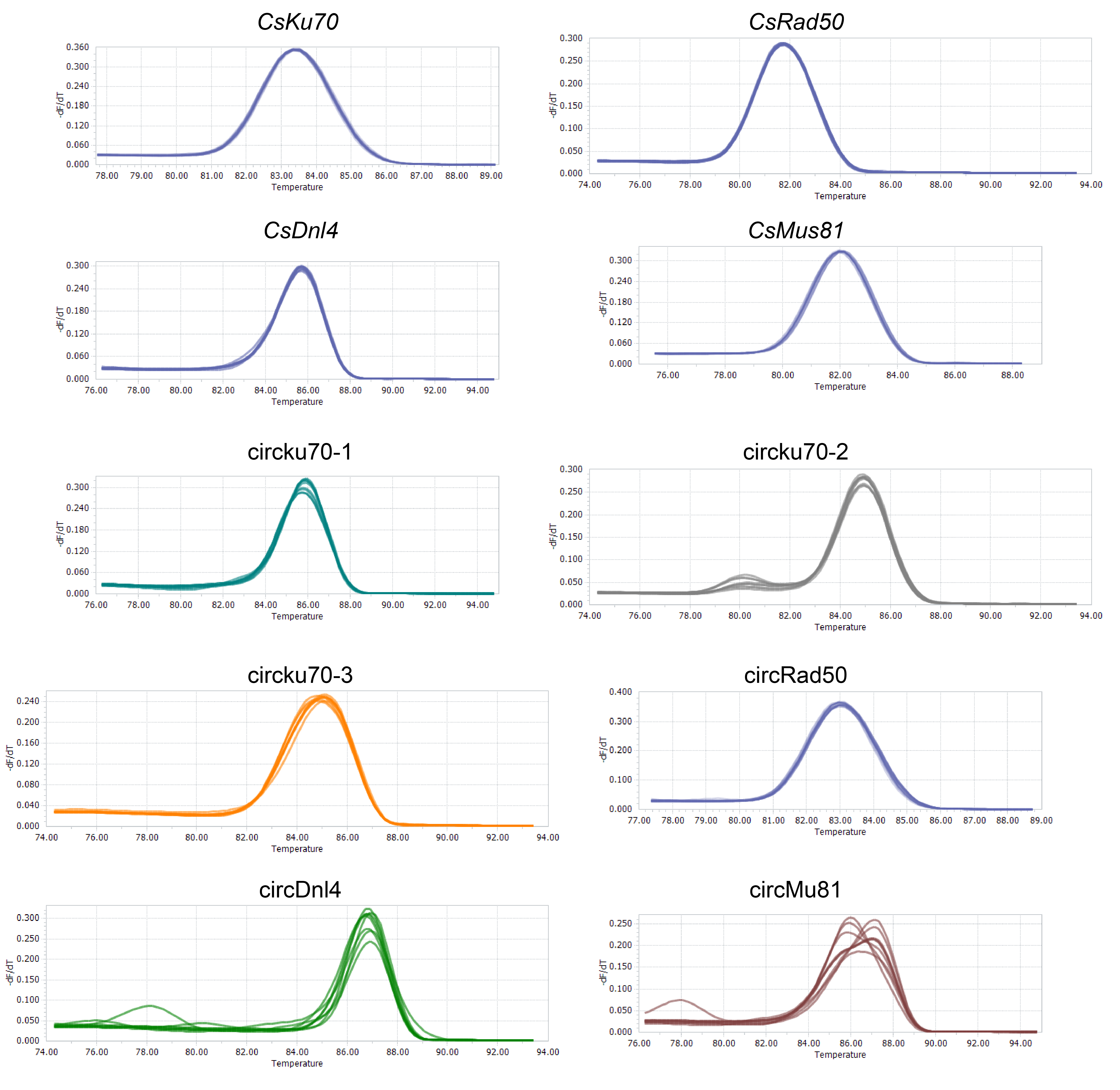

Supplement: Supplementary Figure 4 — Melting curve analysis of qPCR products to verify amplification of a single target in NHEJ and HR pathways. [file Image4.tif]
